# Trehalose Is a Chemical Attractant in the Establishment of Coral Symbiosis

**DOI:** 10.1371/journal.pone.0117087

**Published:** 2015-01-28

**Authors:** Mary Hagedorn, Virginia Carter, Nikolas Zuchowicz, Micaiah Phillips, Chelsea Penfield, Brittany Shamenek, Elizabeth A. Vallen, Frederick W. Kleinhans, Kelly Peterson, Meghan White, Paul H. Yancey

**Affiliations:** 1 Department of Reproductive Sciences, Smithsonian Conservation Biology Institute, Front Royal, Virginia, United States of America; 2 Hawaii Institute of Marine Biology, University of Hawaii, Kaneohe, Hawaii, United States of America; 3 Department of Biology, Swarthmore College, Swarthmore, Pennsylvania, United States of America; 4 Department of Physics, Indiana University-Purdue University Indianapolis, Indianapolis, Indiana, United States of America; 5 Biology Department, Whitman College, Walla Walla, Washington, United States of America

## Abstract

Coral reefs have evolved with a crucial symbiosis between photosynthetic dinoflagellates (genus *Symbiodinium*) and their cnidarian hosts (Scleractinians). Most coral larvae take up *Symbiodinium* from their environment; however, the earliest steps in this process have been elusive. Here we demonstrate that the disaccharide trehalose may be an important signal from the symbiont to potential larval hosts. *Symbiodinium* freshly isolated from *Fungia scutaria* corals constantly released trehalose (but not sucrose, maltose or glucose) into seawater, and released glycerol only in the presence of coral tissue. Spawning *Fungia* adults increased symbiont number in their immediate area by excreting pellets of *Symbiodinium*, and when these naturally discharged *Symbiodinium* were cultured, they also released trehalose. In Y-maze experiments, coral larvae demonstrated chemoattractant and feeding behaviors only towards a chamber with trehalose or glycerol. Concomitantly, coral larvae and adult tissue, but not symbionts, had significant trehalase enzymatic activities, suggesting the capacity to utilize trehalose. Trehalase activity was developmentally regulated in *F. scutaria* larvae, rising as the time for symbiont uptake occurs. Consistent with the enzymatic assays, gene finding demonstrated the presence of a trehalase enzyme in the genome of a related coral, *Acropora digitifera*, and a likely trehalase in the transcriptome of *F. scutaria*. Taken together, these data suggest that adult *F. scutaria* seed the reef with *Symbiodinium* during spawning and the exuded *Symbiodinium* release trehalose into the environment, which acts as a chemoattractant for *F. scutaria* larvae and as an initiator of feeding behavior- the first stages toward establishing the coral-*Symbiodinium* relationship. Because trehalose is a fixed carbon compound, this cue would accurately demonstrate to the cnidarian larvae the photosynthetic ability of the potential symbiont in the ambient environment. To our knowledge, this is the first report of a chemical cue attracting the motile coral larvae to the symbiont.

## Introduction

Symbiotic partnerships between microorganisms and their hosts are critical for the health and function of biological systems ranging from individuals to ecosystems [1]. Most frequently, the basis for the symbiosis is the exchange of nutrients between partners. Symbiotic relationships allow animals and plants to grow in nutrient-poor environments by utilizing the rich metabolic proficiencies of microorganisms. In return, the microbe in these mutualistic partnerships usually gains nutrients and/or a protected environment.

Muscatine and colleagues pioneered an understanding of the marine microalgae-invertebrate association from the late 1950’s onwards [2]. These partnerships allow corals, anemones and sponges to receive fixed carbon from photosynthetic dinoflagellates of the genus *Symbiodinium*. Like other photosynthetic organisms, *Symbiodinium* use the energy from sunlight to reduce CO_2_ into sugar. *Symbiodinium* can release 50–90% of their reduced carbon to their cnidarian host [3–6], providing a critical supply of energy and forming the trophic basis of coral reef ecosystems. The *Symbiodinium* receive a stable location with light for photosynthesis, protection from herbivory and waste products from their host [7].

Although some scleractinian (stony) corals seed their eggs with *Symbiodinium* via vertical infection [8], approximately 85% of all coral host species acquire *Symbiodinium* from their environment, defined as horizontal infection [9], [10]. This usually occurs at the larval stage [11],[12]. While in some instances horizontal infection may be a stochastic event, research in other systems has identified molecules and processes which allow the partners to come into contact, leading to the initiation of the symbiosis. This communication may be bidirectional. For example, in the symbiosis between legumes and *Rhizobia* bacteria, plants first release flavonoids, which cause *Rhizobia* to secrete Nod factor. Nod factors are lipochitin oligosaccharides, which bind to Nod receptors in the plant, leading to the activation of a plant signaling pathway that results in morphological changes and formation of nitrogen-fixing root nodules [13]. In the symbiosis between the Hawaiian bobtail squid *Euprymna scolopes* and the luminous bacterium *Vibrio fischeri*, adults seed the local environment with high numbers of bacteria, and juveniles have specialized tissues to harvest the symbionts from their environment. After infection, signals from *V. fishcheri* induce changes in the squid’s light organ, which hosts the bacteria and most likely prevent colonization or invasion by other bacteria, reviewed in [15].

In the *Symbiodinium*/cnidarian symbiosis, cell-cell recognition for uptake involves lectin-glycan interactions [14], [15]. Surface glycoproteins appear to differ between different *Symbiodinium* clades but the identity of particular molecules involved in recognition is unknown. In the mushroom coral *Fungia scutaria*, the localization of symbionts to particular regions of the larval gastroderm suggests that there may be a set of gastrodermal cells specialized for symbiont uptake [16]. Studies of gene expression during the onset of symbiosis in *F. scutaria* as well as the other scleractinian corals *Acropora palmata* and *Montastraea faveolata* has shown few detectable transcriptional changes upon infection with appropriate symbionts [17,18]. After uptake, the *Symbiodinium* are maintained intracellularly in a membrane-bound compartment called the symbiosome. Recent work with the sea anemone *Aiptasia pallida*, a model system for the cnidarian-*Symbiodinium* symbiosis, has documented extensive transcriptional differences between established symbiotic and aposymbiotic (lacking symbionts) animals demonstrating the many long-term alterations in gene expression that result from changes in host physiology as a result of the symbiosis [19].

This report focuses on one of the earliest steps in establishment of the cnidarian-*Symbiodinium* symbiosis: how the partners find and attract one another. A previous report on the onset of symbiosis in *F. scutaria* demonstrated that symbiont uptake was increased by the presence of an externally-added feeding stimulus, homogenized *Artemia* (brine shrimp), but did not depend on it, suggesting that the symbiont may have stimulated a feeding response but this was not investigated further [12]. Other studies on the sensation and detection of the partners focused on a larval-produced attractant for the symbiont. For example, data from Horiguchi et al. [20] and Hollingsworth et al. [21] support a “beacon hypothesis” where motile *Symbiodinium* specifically swim toward light generated by green fluorescent proteins on the mouthparts of cnidarian larvae. Other experiments describe chemical attractants [22], such as nitrogen-based cues [23]. In contrast, a demonstration of signaling from the symbiont to the animal partner comes from the freshwater system, where the symbiont of the cnidarian hydra produces maltose to attract the host [24], [25].

The objective of this study was to determine whether *Symbiodinium* synthesize and excrete chemoattractants sensed by cnidarian larvae. Two recent reports demonstrate that coral, crustacean, and cephalopod larvae as well as juvenile reef fish prefer water from healthy reefs, suggesting that chemical cues sensed by these organisms play a role in recruitment and settlement [26], [27]. We examined both the production and excretion of sugars from *Symbiodinium*, and sugar levels in adult coral from three species and developing larvae from one species. To determine whether the coral larvae would follow chemical cues, we tested their chemo-orienting and onset of feeding behaviors when placed in a Y-maze and presented with potential chemo-attracting sugars. The final set of experiments focused on analysis of enzymes that might break down the sugar within the *Symbiodinium* and coral, as well as analysis of the developmental expression of these enzymes.

Three species of coral were used in these experiments, with the majority of the data collected from individual adult polyps of *F. scutaria* and their larvae. Comparative data was also collected from adult *Porites compressa* and *Pocillopora damicornis* fragments to determine whether members of different scleractinian families behaved similarly to *Fungia. F. scutaria* is a useful model organism for these studies, because its spawning, development [28] and symbiotic onset [11] are well described, and occur at least 4 times throughout the summer months (following the full moon) [28]. Uptake of symbionts in *F. scutaria* occurs around 3 to 5 days post-fertilization [12]. Within a few more days, the larvae change shape, becoming more spherical, and stop swimming and begin crawling before settling and undergoing metamorphosis (with approximately 50% of larvae having settled by day 7) [12]. The uptake of *Symbiodinium* may aid in metamorphosis and settlement, but this appears to be somewhat variable [12].

Our array of experiments focusing on defining the chemoattractants excreted by *Symbiodinium* and identifying the host enzymes responsible for sugar degradation, coupled with suggestions about how this might work on the reef, support the hypothesis that trehalose is a chemical attractant in the establishment of coral symbiosis.

## Methods

### Coral specimens

Corals or coral fragments of *F. scutaria, P. compressa* and *P. damicornis* were collected from various shallow reef flats around Coconut Island in Kaneohe Bay, Hawaii, and then maintained in shallow running seawater tables at the Hawaii Institute of Marine Biology, University of Hawaii, Coconut Island. Collection was performed with the appropriate permits from the state of Hawaii’s Department of Land and Natural Resources (Special Activity Permit # SAP 2011-1 and SAP 2012-63). *Symbiodinium* were extracted and coral larvae were produced following the methods of Hagedorn et al. [29] and [30], respectively. All captive and wild *F. scutaria* coral were individually tagged.

A subset of the 60 to 80 adult *F. scutaria* coral in seawater tables spawned during a two to three night period during the summer months (June to September) over three years. The larvae for the described experiments were generated and reared in bowls cleaned daily, consisting of eggs from one individual with sperm from 5 to 7 males. For experiments, thousands of larvae were collected from at least 3 to 5 different bowls to accommodate any slight developmental differences in the bowls. Only one such pooled sample was collected on each developmental day, so the ‘n’ for larvae indicates the number of different pooled larval samples. For *Symbiodinium*, the ‘n’ indicates the number of individual corals the algae were extracted from or the number of pellets of naturally excreted *Symbiodinium* either from captive or wild coral. For adult coral, the ‘n’ indicates the number of discrete individuals, unless otherwise stated. The sample size for each experiment is defined in each figure legend or table.

### Tissue preparations

***Fungia scutaria* coral larvae**. Coral larvae (without symbionts) were concentrated in 40-μm cell sorting baskets (Falcon, Fisher). Larvae used for enzyme and internal sugar assays (methods described below) were blotted through the mesh to remove excess seawater and the larval concentrate (20,000 to 40,000 larvae/tube) was transferred into 1.5 ml microcentrifuge tubes and rapidly frozen in liquid nitrogen to −196°C, before storage at −80°C until further processing.

**Adult coral tissue**. Enzyme levels and internal sugars were measured from small coral pieces (~2cm square, n = 5 individuals from each species) from three species, *F. scutaria, P. damicornis* and *P. compressa*. Pieces were flash frozen in liquid nitrogen and airbrushed to remove tissue from the skeleton into a Ziploc bag, and then squeezed into a preweighed microcentrifuge tube.

**Extracted and naturally excreted *Symbiodinium***. *Symbiodinium* were extracted from adult coral tissue with the following methods. Adult coral tissue (tissue/*Symbiodinium* complex) was dissociated from the skeleton with a water pik following the methods of Hagedorn et al. [29]. Adult tissue was collected into a large beaker, moved to 50 ml plastic centrifuge tubes, and then centrifuged at 3,200 × *g* for 15 minutes to remove excess water. The tissue was homogenized using a glass homogenizer, and resuspended into filtered seawater. Samples were then centrifuged at 9,800 × *g* for 10 minutes, causing *Symbiodinium* to pellet at the tube bottom. In the first cleaning, the coral tissue layer was removed from the top of the *Symbiodinium* pellet. In some experiments when a cleaned coral tissue stimulus was needed, this layer was saved. Otherwise it was discarded and the *Symbiodinium* were centrifuged 3 to 4 times to further clean them and then used in various experiments.

Naturally excreted *Symbiodinium* were isolated from *F. scutaria* pellets produced around spawning from both captive and wild animals. Captive animals were observed for 3 to 4 days before, during and after spawning. The wild animals were observed on the reef off of Coconut Island for 7 consecutive days in August 2013 during and after the spawn. Pellets were collected using 5 ml transfer pipettes and placed into 1.5 ml microcentrifuge tubes, removing excess seawater and topping to 1000 μl with FSW (filtered seawater) and homogenized using a microcentrifuge sample pestle to disperse the pellet without shearing the symbionts. Following the methods of Hagedorn et al. [29], a Walz Junior pulse amplitude modulation (PAM) Chlorophyll Fluorometer was used to measure the functionality of photosystem II in the expelled *Symbiodinium* as a indicator to ensure there were live symbionts within the pellet. This was performed with all pellets isolated from the captive animals. However, because the samples collected on the reef were contaminated with sand and algal debris from wave action, PAM values were not acquired beyond the first night of collection for pellets obtained in the wild. *Symbiodinium* in each sample were counted with a hemocytometer on an Olympus BX41 at 200x under both white and fluorescent lighting, using a 450 to 480 nm band pass filter. If the sample demonstrated glowing red cells of the correct size and shape to be *Symbiodinium* under fluorescent lighting and a PAM quantum yield above 0.15, the sample was scored as containing live *Symbiodinium*, and processed as described above.

**Weight and dry weight measurements and calculations**. The percent wet and dry weight was measured for adult coral tissue and coral larvae and *Symbiodinium* to allow us to normalize values for sugars and enzymes across tissues and species for more accurate comparisons. Some measurements were taken from previous work, as noted in the table or figure. The wet weight of adult coral tissue was determined from small pieces of coral (~2cm square, n = 5 individuals from each species), of *F. scutaria, P. damicornis* and *P. compressa* that were flash frozen in liquid nitrogen. Each piece was then airbrushed to remove tissue from the skeleton into a Ziploc bag, squeezed into a preweighed microcentrifuge tube, reweighed to determine the wet weight, and processed as described below. The wet weight of developing coral larvae was measured from thousands of concentrated larvae collected in 40-μm cell sorting baskets (as described above) on each developmental day. Then, the larvae were placed into preweighed 1.5 ml microcentrifuge tubes (n = 4 to 5 tubes/developmental day). The tube was reweighed to determine the wet weight and processed as described below. The wet weight for the *Symbiodinium* from each adult coral species was determined from extracted symbionts (n = 5 individuals from each species of the 3 species) that were placed in preweighed microcentrifuge tube, filled with cells (~ 2 × 10^6^/ml), centrifuged at 8,100 × g, excess water decanted, the pellet dried by blotting with a Kimwipe, reweighed to determine the wet weight and processed as described below.

To determine the dry weight of all tissue types, samples were placed into a drying oven at 60°C for 3–7 days. At the end of the drying period, the microcentrifuge tubes were closed and immediately weighed again. Once the weight of the tube was subtracted, the percent dry weight was calculated as (g dry weight/ g wet weight) × 100. Microcentrifuge tubes without any tissue were also weighed, left in the oven and reweighed as control for changes in the tubes during the drying period. No change in weight was noted in the empty tubes during this process. For calculations of enzyme activities and solute contents per dry weight, the actual grams dry weight were used because the amount of water held in different tissues varied.

### Assessment of potential sugar chemoattractants

**Manipulations of *Symbiodinium***. Excreted sugars were measured from extracted or naturally expelled *Symbiodinium* as described [31] with some modification as noted below. *Symbiodinium* were diluted to 2×10^6^ cells/ml in 1.5 ml plastic microcentrifuge tubes, and then mixed 1:1 with either filtered seawater (FSW), FSW and coral tissue (~ 50 μl fresh or previously boiled for 10 min at 100°C tissue [32], 7 vials/treatment) or FSW with ~1,000 live Day 1 *F. scutaria* larvae (n = 4), resulting in a one ml final volume. Thousands of larvae from each sample were concentrated in a 40-μm cell-sorting basket before use. We used Day 1 larvae to reduce the possibility that the larvae might consume the trehalose. Samples were incubated in microcentrifuge tubes at 24 to 25°C in a flowing seawater table to maintain temperature under natural sunlight for 1 h. The *Symbiodinium* samples were spun at 9,800 × *g* for 10 minutes. The supernatant was removed and the remaining pellet and separate supernatant were rapidly frozen in liquid nitrogen. To analyze physiological processes, Muscatine [33] used radioactive bicarbonate and a 100% ethanol extraction followed by two-dimensional chromatography for analysis, instead we used a 70% ethanol extraction and analyzed the symbionts’ seawater for trehalose and glycerol using enzymatic assays [34] (see also below).

**Measurement of sugars present in coral, coral larvae, *Symbiodinium* and seawater**. Frozen tissue aliquots were homogenized in 70% ice-cold ethanol, with proteins allowed to precipitate overnight; then the homogenates were centrifuged at 15,000 rpm for 30 min and the supernatants were run overnight in a rotary evaporator to remove the ethanol. The evaporates were dissolved in 600 μl pure water.

Samples were analyzed by spectrophotometry utilizing enzymatic assays. Kits and reagents were all from Sigma-Aldrich and used according to the manufacturer’s directions. Glycerol was measured with reagent #F6428, sucrose with kit #SCA-20 and maltose with kit #MAK018 containing acid alpha-glucosidase (a maltase). Trehalose was analyzed with pig-kidney trehalase following the methods of Sigma bulletin SSTREH01. Trehalose and maltose are both disaccharides composed of two glucose molecules. Therefore, the product of enzymatic digestion of one of these sugar molecules is two glucose molecules. The glucose produced by the trehalase and maltase enzymatic assays was measured using a hexokinase glucose assay (Sigma-Aldrich #G3293) with the amount of trehalose or maltose present originally corresponding to half the glucose found after cleavage. To test for glucose already present in the tissues, extracts were measured without the addition of the hydrolyzing enzymes (trehalase, etc.).

**Chemotaxis assays**. Chemo-orienting behavior of coral larvae to a variety of sugars was measured in a Y-maze. A chamber was created using a glass slide with two stacked pieces of electrical tape (~ 600 μm total finished thickness) with a Y-shape cut out in the center of the tape. A total volume of 250 μl of 0.2 μm filtered seawater was added to the maze, then 20 μl of seawater containing larvae (100 to 500/run, pooled from at least 5 bowls) were added to the base of the Y, and finally 0.5 μl volume of a solution (0.1 M sucrose, trehalose, maltose, glycerol or glucose or seawater) was added to one arm and 0.5 μl of 0.2 μm filtered seawater was added to the other. To determine whether the larvae could respond to lower concentrations of trehalose in the maze, 0.01 and 0.001M solutions were also tested. The arms containing the test solutions were randomly changed. All test solutions, as well as the control seawater, contained 1 mg/ml methylene blue (Sigma, St Louis, MO) to visualize the movement of the solute front.

Two types of runs were examined: 1) *test-runs* had one arm with a sugar solution added (n = 10 to 20 runs/solution) and the other arm with seawater added, and 2) *control-runs* had seawater in both arms (n = 10 to 20 runs/solution). The larvae were allowed to swim for 3 min, photographed, and then the larvae present in each arm were counted. This area was chosen because it was the limit of visual detection of the dye after 3 min. To compare the responses, the percent difference was calculated as (a−b)/((a+b)/2)*100, where a and b are the numbers in each arm, and then the numbers were translated with a constant and then log (10) transformed.

**Measurement of enzymes present in coral, coral larvae and *Symbiodinium***. Activities of trehalase, sucrase and maltase were measured in weighed aliquots (~0.2 g) of frozen aposymbiotic larvae, adult tissue (with symbionts within the tissue) or extracted symbionts. All tissue was homogenized in 0.01M potassium phosphate buffer pH 7.4 (with a 1:4 ratio of coral tissue to phosphate buffer). The reaction mixtures contained 600 μl of 0.1M potassium phosphate buffer (pH 7.4) containing 20 mM trehalose, sucrose, or maltose with 150 μl tissue homogenate, and were incubated at 30°C for 60 min. The tube was then boiled for 5 min to denature enzyme activity, cooled, and centrifuged for 10 min at 15,000 rpm. Then, 100 μl of the supernatant was assayed for glucose in 1.00 ml of a spectrophotometric hexokinase glucose assay (Sigma-Aldrich #G3293). We report the specific activity, expressed in units of μg glucose generated/h/g dry weight. To test for glucose already present in the tissues, reaction mixtures were incubated without added disaccharides, boiled, and then tested for glucose; those values were subtracted from the glucose measured with the disaccharides. Disaccharides were also added to extracts after boiling to test for glucose contamination in the disaccharide reagents, which was found to be undetectable.

**Identification of potential trehalase-encoding genes in a coral**. Sequences from *Drosophila melanogaster* (*D.m.*AAM68193.1) and *Saccharomyces cerevisiae* (*S.c.* YDR001C) were utilized as queries to search *Acropora digitifera* genome sequences hosted at http://marinegenomics.oist.jp/genomes/gallery?project_id=0 using tblastn. *A. digitifera* was used as it represents the most complete coral genome available and, like *F. scutaria* acquires its *Symbiodinium* from the environment [35], [36]. The *A. digitifera* sequence with significant homology to the query sequences was examined using Prosite (prosite.expasy.org).

**Statistics**. All data analyses in this study were performed using Instat 3.0 or Prism 5.0 (Graphpad, San Diego, CA) and Excel (Microsoft, Redmond, WA). For multiple group comparisons, normality was tested graphically and ANOVAs were done with a Tukey’s Multiple Comparison Test or Student-Newman-Keuls post-test. All percentage data was log transformed prior to ANOVA analysis. These tests are identified in the results when reporting the P-value, and those values ≤ 0.05 were considered significant.

## Results

### Extracted and naturally-expelled *Symbiodinium* excrete trehalose

**Figure 1:**
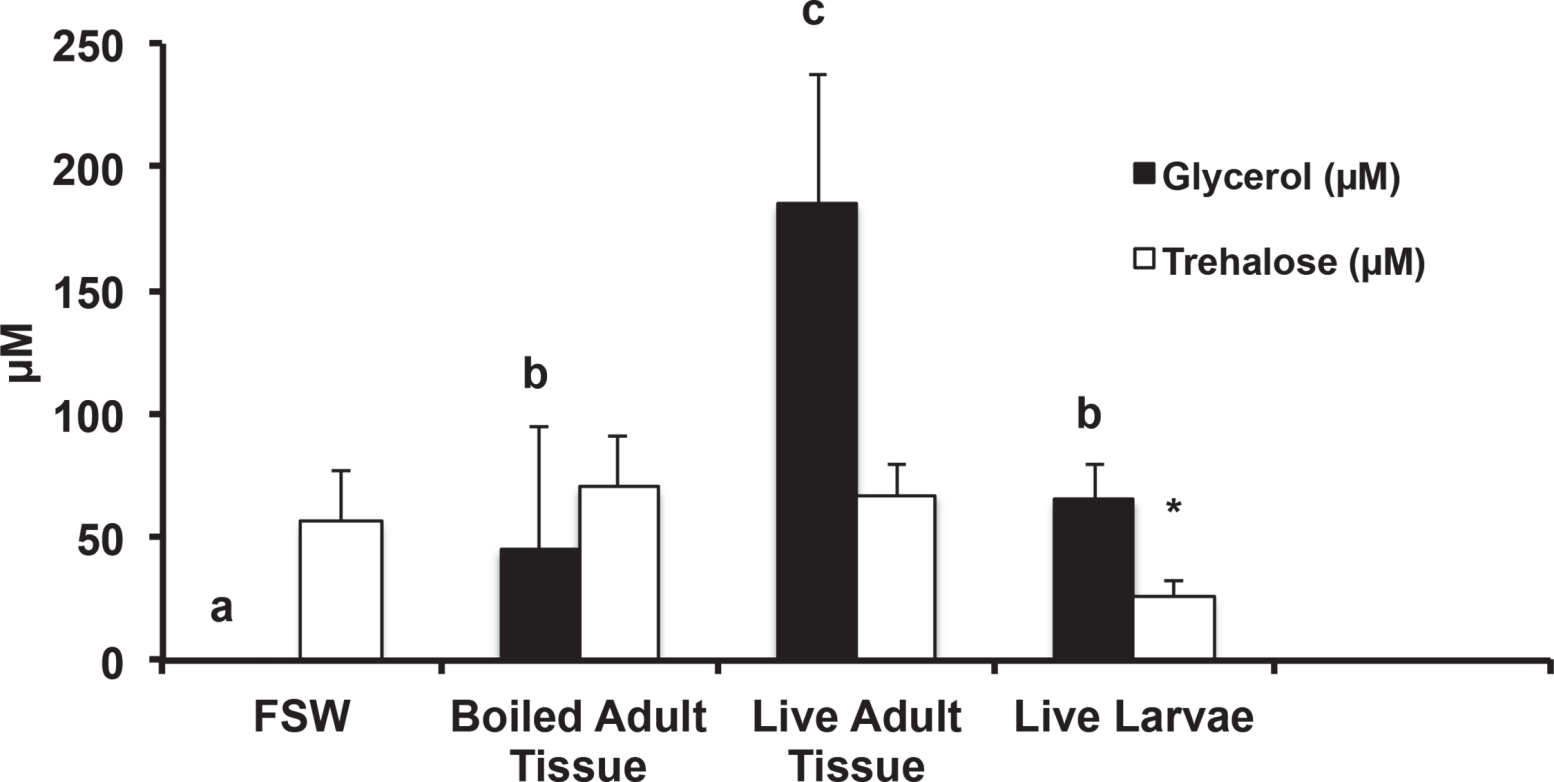
Trehalose is constitutively excreted by *Symbiodinium* isolated from *F. scutaria*, while glycerol is not. The amounts of glycerol and trehalose excreted by *Symbiodinium* were assayed enzymatically. *Symbiodinium* were incubated with filtered seawater (FSW; n = 7 individual coral), boiled adult coral tissue (n = 7 individual coral), live adult coral tissue (n = 7 individual coral) or swimming coral larvae (n = 4 individual coral, samples had ~ 1,000 swimming larvae in 1ml) for 1 h. *Symbiodinium* co-cultured with the live larvae (*) yielded lower trehalose (P < 0.05 to < 0.01 versus all other groups), perhaps due to consumption by the larvae. Pure FSW without symbionts had no detectable trehalose or glycerol. The letters a, b, c and * indicate statistical differences (ANOVA, Student-Newman-Keuls post-test; see P values above).

It seemed most likely that a fixed carbon compound would be a chemoattractant synthesized by *Symbiodinium*. Such a compound would be feasible for the symbiont to make and would reflect photosynthetic ability under the ambient environmental conditions. Therefore, to determine what sugars *Symbiodinium* synthesized and excreted as possible attractants, we repeated and extended Muscatine’s 1967 [33] experiments. He found that about 40% of the *Symbiodinium’s* photosynthate (produced in 1 h) was liberated as glycerol in response to the presence of the host coral tissue, but found no glycerol release when the tissue was absent or boiled. We saw a similar change in glycerol excretion when tissue-extracted *Symbiodinium* were incubated with host coral tissue. Strikingly, however, we found that trehalose was released constitutively, without the need for co-incubation with the host tissue (Fig. 1). Our analysis shows lower levels of trehalose in the presence of coral larvae compared to other samples, but it might be that trehalose levels in these samples are somewhat decreased by the uptake and utilization of trehalose by the larvae in those samples.

We believe that no one has previously reported trehalose as important in the *Symbiodinium*/cnidarian symbiosis previously because: i) the levels released from *Symbiodinium* in 1 h incubations are relatively low; ii) it may not be produced from radioactive carbonate during photosynthesis during incubations using tracers in 1 h; and iii) 100% ethanol was used for paper chromatography of sugars in Muscatine’s 1967 experiments [33], and trehalose can crystallize out of ethanol solutions at 80% or higher concentration [37], thus removing it from consideration by this process.

To obtain a more detailed profile of sugars released from *Symbiodinium* in a variety of different subclades, we used freshly isolated *Symbiodinium* from *F. scutaria, P. compressa and P. damicornis*. These symbionts are all *Symbiodinium* clade C, but belong to different subclades [38]. *Symbiodinium* isolated from the cnidarian host (10 ml at 10^6^ or 10^7^ cells/ml in FSW) were incubated in sealed vials at 24 to 25°C under natural sunlight for 6 h (*F. scutaria* symbionts) or 24 h (all others). During this incubation, *F. scutaria Symbiodinium* released trehalose, which accumulated to 650 μM (Table 1). In contrast, far less sucrose, maltose, and glucose were released (Table 1). While the numbers of symbionts and the time of incubation are different in these experiments, the same pattern was found for *Symbiodinium* isolated from the two other coral species (Table 1), suggesting this may be a broad physiological trait of cnidarian symbionts. Using the measurements from *F. scutaria’s Symbiodinium* (e.g., 650 μM from 10^7^ cells/ml in 6 h; Table 1), we estimated that each *Symbiodinium* cell released ~1 ×10^−14^ moles of trehalose/h.

**Table 1:** *Symbiodinium* Excretion of Disaccharides and Glucose into FSW.

**Source of *Symbiodinium***	**Trehalose, μM**	**Maltose, μM**	**Sucrose, μM**	**Glucose, μM**
*F. scutaria* (6 h, 10^7^/ml; n = 4)	650 ± 90	24 ± 18	53 ± 16	< 9
*P. compressa* (24 h, 10^6^/ml; n = 3)	461 ± 144	< 5	89 ± 9	< 9
*P. damicornis* (24 h, 10^6^/ml; n = 3)	422 ± 119	< 5	59 ± 9	< 9

n = number of individual coral the *Symbiodinium* were extracted from.

Having established that *Symbiodinium* can release trehalose into the environment when freshly isolated from host tissue, we next asked whether *Symbiodinium* naturally released by *F. scutaria* during their reproductive phase also excreted trehalose. To answer this, we cultured naturally released pellets (n = 5) in 1.0 ml FSW for 6 h in sunlight. Their trehalose production, analyzed in the FSW after the cells were removed by gentle centrifugation, was 12.2 ± 10.7 μM (SD). On average 429,000 cells/ml (± 249,000 SD) within the pellets were scored as live *Symbiodinium* (defined in Methods). Using the value from Table 1 (650 μM trehalose from 10^7^ cells/ml over 6 h), the average 429,000 live cells in the pellets should have produced an estimate of 29 μM trehalose over 6 h.

To understand more fully the sources and levels of trehalose and other sugars in the symbiont/coral complex, we analyzed symbionts, larvae and adult tissues for internal disaccharides, as well as glycerol and glucose. *F. scutaria* symbionts contained a wide variety of sugars within their cells, including trehalose, glucose, and glycerol (Table 1 and Yancey et al. [34]). Aposymbiotic *F. scutaria* larvae contained trehalose and glucose in relatively constant, low amounts throughout the developmental period studied, and adults contained similar levels of glucose, but higher levels of trehalose and glycerol (Table 2). We do not know whether the trehalose found in the symbiotic adult tissue is made by the symbionts, cnidarians or associated bacteria. Similarly, the low levels of trehalose in the aposymbiotic larvae could be synthesized by the larvae, maternally provisioned, or from bacteria. Sequence analysis of various cnidarian databases did not identify trehalose synthetases that were clearly of animal origin, as the sequences identified were most closely related to protist sequences and may be due to contamination with *Symbiodinium* DNA (data not shown).

**Table 2:** Internal Sugar and Glycerol Contents of Tissues (μmol/g dry wt).

**Tissue Source**	**Internal Trehalose**	**Internal Glycerol** ^†^	**Internal Glucose**
*F. scutaria Symbiodinium*	7.20 ± 2.01 (n = 4)	10.1 ± 4.10 (n = 7)	2.49 ± 0.64 (n = 4)
Adult *F. scutaria* *	8.25 ± 0.21 (n = 3)	5.53 ± 4.08 (n = 7)	1.84 ± 1.15 (n = 3)
Adult *P. compressa* *	8.71 (n = 1)	4.00 ±1.88 (n = 7)	0.950 (n = 1)
Adult *P. damicornis* *	7.47 (n = 1)	3.93 ± 3.21 (n = 7)	0.960 (n = 1)
Larval Day 1**	2.00 ± 0.55 (n = 4)	nd (n = 4)	0.880 ± 0.570 (n = 4)
Larval Day 3**	2.46 ± 0.13 (n = 3)	nd (n = 4)	0.690 ± 0.420 (n = 3)
Larval Day 4**	2.97 ± 0.71 (n = 4)	nd (n = 4)	0.830 ± 0.190 (n = 4)

† Non-larval glycerol values from Yancey et al. [34]

Nd Not detected (threshold for detection is approximately 0.10 μmol/g dry wt)

* With symbionts.

** Aposymbiotic *F. scutaria* larvae.

Dry weights were derived as described in Methods.

### Coral larvae are attracted to trehalose

**Figure 2:**
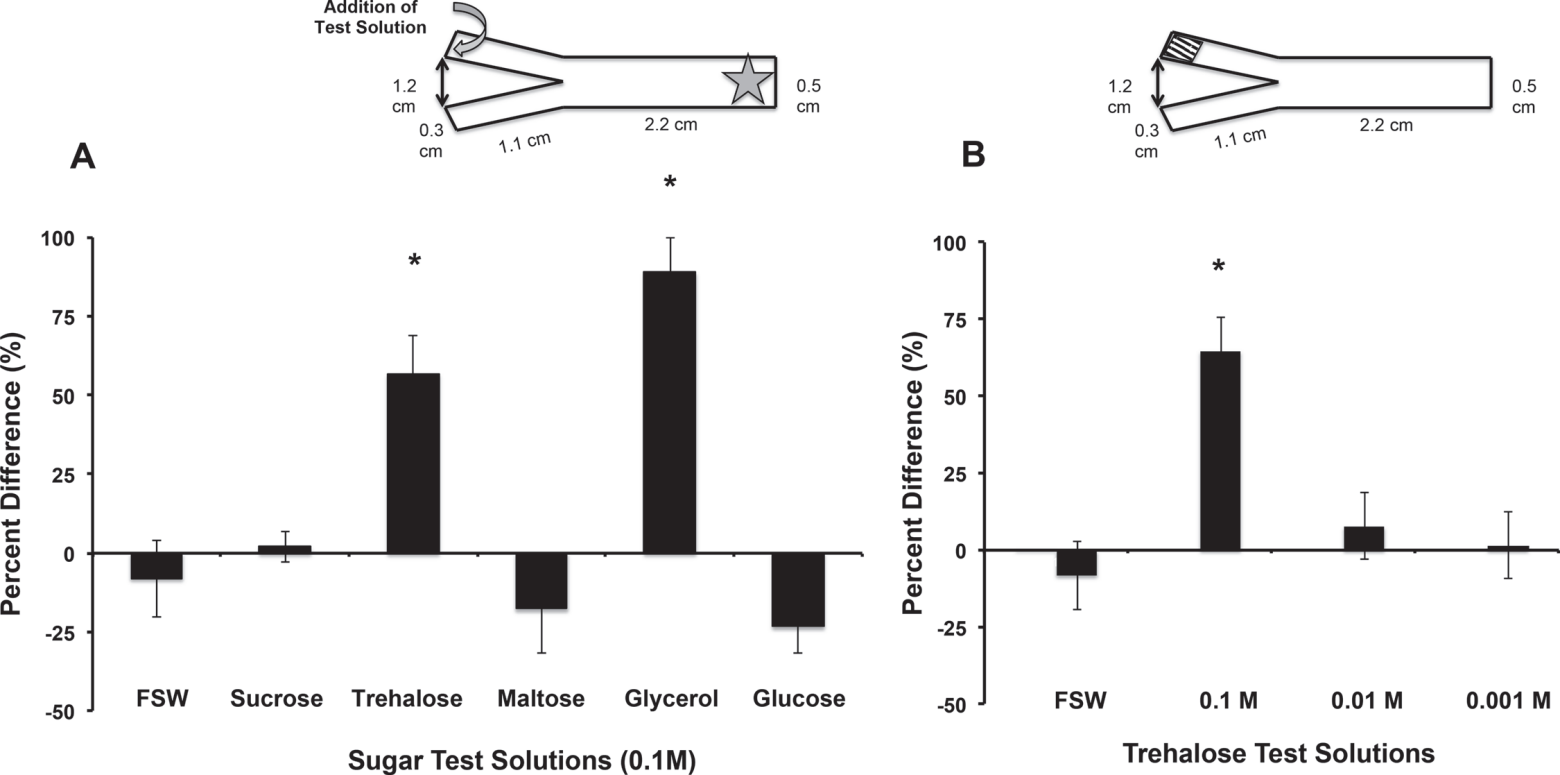
Trehalose and glycerol are chemo-attractants for *F. scutaria* larvae. *F. scutaria* larvae were placed in a Y-maze, and their orienting behavior monitored. The test solutions were introduced to the maze at the ends of each arm, indicated by the curved gray arrow, while the larvae were added at the base, as indicated by the star (inset A). **A)** Only glycerol and trehalose solutions demonstrated clear chemo-orienting behavior different from the controls (*P < 0.05, ANOVA, Tukey’s Multiple Comparison test) while the other solutions remained neutral (P > 0.05, ANOVA, Tukey’s Multiple Comparison test). **B)** Only the 0.1 M trehalose demonstrated clear chemo-orienting behavior different from the 0.01 and 0.001M trehalose solutions and controls (*P < 0.05, ANOVA, Tukey’s Multiple Comparison test).

Given that *Symbiodinium* excrete trehalose, we asked whether it might play a role in attracting coral larvae for the initiation of symbiosis. To test this, we placed Day 3 *F. scutaria* larvae without symbionts in a Y-maze and tested whether they were attracted to specific sugars (Fig. 2A). The larvae showed distinct orienting responses only to glycerol and trehalose (P <0.05, ANOVA, Tukey’s Multiple Comparison test) while the other solutions were neutral (P >0.05, ANOVA, Tukey’s Multiple Comparison test), and a 0.1M trehalose solution was the lowest concentration that produced this response (Fig. 2B). In these experiments with a 0.1 M trehalose solution, there was an obvious accumulation of larvae in the arms where trehalose or glycerol was added. Their selective behavior is demonstrated in supplementary video clips. Specifically, for sugars other than trehalose or glycerol, and for control (FSW) treatments, the larvae swam constantly with no clear pattern to their behavior, and they did not change their morphology during the trial (S1 Fig.). However, when the larvae discovered the trehalose or glycerol solutions, they stopped swimming, changed their morphology from elongated to more spherical, settled to the bottom of the maze, and began feeding behavior (S2 Fig.).

The spot where the larvae stopped swimming, sank to the bottom and started feeding was at a point 2 mm from the end of the maze, indicated by the shaded area of inset in Fig. 2B. This volume is approximately 10 μl. Thus, if 0.5 μl of a 0.1M trehalose solution were to fully mix in this volume in 3 min, a rough upper estimate of the coral larvae’s sensitivity to trehalose would be 5 mM.

Because the *Symbiodinium* released glycerol only when in contact with host tissue, it is unlikely to function as a chemoattractant during the initiation phase of symbiosis. In contrast, *Symbiodinium* release trehalose constitutively and coral larvae are attracted to it, suggesting it might play a role in the initiation of symbiosis.

### Trehalase is present in corals and developmentally regulated in coral larvae

We measured activities of trehalase and other disaccharidases in the developing *F. scutaria* larvae, as well as in adult coral tissues and in isolated symbionts to determine whether these organisms had the capacity to metabolize these sugars. To compare activities among the extracts, which varied widely in water content, we normalized the data with tissue dry weights following the methods of Hagedorn et al. [39], [29] and Yancey et al. [34].

**Figure 3:**
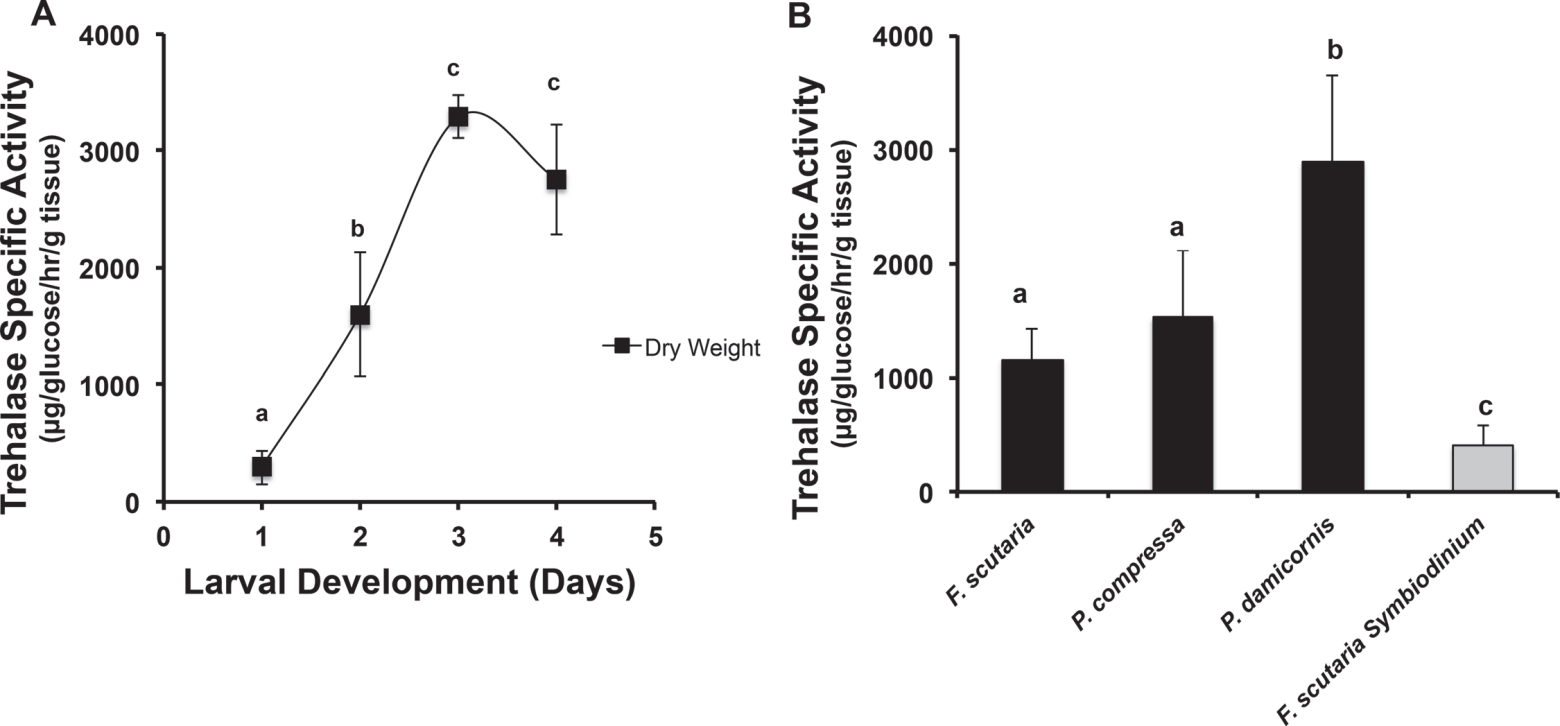
Coral larvae and adults contain trehalase activity. The mean specific trehalase activity was calculated by determining how much exogenously added trehalose was cleaved into glucose (in units of μg glucose/h/g dry or wet weight ± SD). **A)** Trehalase from *F. scutaria* larvae is per dry weight; a, b, c, d indicate statistical differences at *P* < 0.01 (ANOVA, Student-Newman-Keuls post-test, n = 3 to 5 with thousands of larvae/assay from each developmental time point). The highest activities correspond to the time point when coral larvae are first capable of symbiont uptake and may be actively seeking them, at Days 3–4. **B**) Adult coral tissue from 3 different coral species (black bars) and one *Symbiodinium* species (n = 3 individuals from each coral species, n = *Symbiodinium* from 5 *F. scutaria* individuals, grey bar) all contain trehalase activity; a, b, c indicate statistical differences (ANOVA). The highest trehalase activity was in *P. damicornis*, significantly higher (P < 0.05) than the other corals; and activity was found to be significantly lower in the *Symbiodinium* compared to all corals (P <0.01 to 0.05, Student-Newman-Keuls post-test), suggesting the dinoflagellates may only metabolize this sugar at low rates.

In *F. scutaria* larvae, we found that the trehalase and sucrase enzymes were developmentally regulated and strikingly increased over time. The specific activity of trehalase, expressed in units of μg glucose generated/h/g dry weight (Fig. 3A), was low in Day 1 larvae (294 ± 146 SD units), but rose 11-fold to peak at Day 3 (3,291 ± 181 SD units). This is especially noteworthy because Day 3 has been previously identified to be the first time that *F. scutaria* larvae are capable of uptake of *Symbiodinium* [12]. Sucrase activity also increased during this same developmental period but had lower overall values during the entire period (38.9 ± 7.4 SD units at Day 1, peaking at 321 ± 80 SD units at Day 3). Nevertheless, both enzymes displayed a greater than 10-fold increase at Day 3 over their initial Day 1 values. While the apparent maltase activity was considerably higher than trehalase, it was without a dramatic rise during development (10,873 ± 254 SD units at Day 1; 15,747 ± 1305 SD units at Day 3), displaying only a 1.5-fold increase at Day 3 over their initial Day 1 values. It is important to note that the activity measured as “maltase” may be a less specific enzyme that also hydrolyzes large glucose polymers stored in the larvae. For example, mammalian acid maltase and alpha-glucosidase not only hydrolyze maltose but also larger glucose polymers [40] and the high levels of enzyme activity may reflect the catabolism of these polymers in the larvae.

Intact adult coral tissue (containing *Symbiodinium*) from all three species tested also contained substantial levels of trehalase activity, ranging from 1,159 ± 263 to 2,891± 752 SD units (Fig. 3B). *F. scutaria* adults had about 35% of the peak activity of the larvae.

However, when *F. scutaria’s Symbiodinium* were separated from their coral host and examined, they had much lower levels of enzyme activity (407 ± 173 SD units per g dry weight; Fig. 3B), corresponding to less than 15% of the peak larval activity and 35% of the adult *F. scutaria* activity. Therefore, *Symbiodinium* cells contained and excreted substantial concentrations of trehalose but had low levels of the enzyme to break it down, suggesting that this sugar may be synthesized more for export than as a source of internal energy.

## Discussion

### Trehalose as a Chemoattractant

Based on several lines of evidence presented here, we believe that trehalose produced by *Symbiodinium* may be a cue to help attract *F. scutaria* larvae for initiation of symbiosis. First, trehalose was produced and excreted by recently excised and wild *Symbiodinium* without the need of a host factor. Second, *Fungia* larvae followed both a glycerol and trehalose gradient, but as glycerol was only released by *Symbiodinium* in close proximity to live coral tissue, this cue is unlikely to play a role in the orienting events. Moreover, recent evidence indicates that glycerol release may be a stress response in *Symbiodinium* [41]. In contrast, it is unlikely that the excretion of trehalose into the environment by *Symbiodinium* is a symptom of stressed, lysed or dead cells. If the cells were lysed or dying the ratio of internal and external sugars should be similar. However, the ratio of excreted and internal trehalose and glucose in the *Symbiodinium* samples are quite dissimilar (Tables 1 and 2). Additionally, the *Symbiodinium* demonstrated a differential response to the presence of coral tissue for glycerol excretion (Fig. 1), which was not observed for trehalose excretion. Taken together, these data suggest that the *Symbiodinium* cells we have assayed have active metabolism and are regulating their physiology. Third, both adult and developing coral larvae produced the specific enzyme needed to break down trehalose, and the concentration of the enzyme increased during the developmental period when *F. scutaria* is taking up its symbionts (Days 3 and 4). Moreover, even though the symbionts produce trehalose, they produce very little of the trehalase enzyme.

BLAST analysis identified a likely trehalase enzyme from a scleractinian coral, supporting the enzymatic assay data described above demonstrating cnidarian trehalase activity. We used the fruit fly (*Drosophila melanogaster)* and budding yeast (*Saccharomyces cerevisiae)* trehalase sequences as queries. Sequences from these organisms were used because trehalase enzyme activity has been mapped to a specific genetic locus in both, demonstrating unequivocally these genes encode trehalases, and not related disaccharidases [42,43]. We searched the published *Acropora digitifera* genome (http://marinegenomics.oist.jp/genomes/gallery?project_id=0), another scleractinian coral that takes up symbionts at its larval stage [44]. A single match with significant homology to both *D. melanogaster* and *S. cerevisiae* was found in both organisms. The *A. digitifera* sequence was found on scaffold 6518:25988-27781 and was 37% identical and 54% similar to the *D. melanogaster* protein throughout the entire length of the protein (S3 Fig.). Further analysis confirmed the *A. digitifera* sequence had 100% identity with the signature motifs of trehalase enzymes as defined by Prosite (http://prosite.expasy.org/PDOC00717) (S3 Fig.). Additionally, a search of *F. scutaria* transcriptome data produced sequence with significant homology to trehalase enzyme in *A. digitifera* (S4 Fig.).

Taken together, these data show that coral larvae and corals have the capacity to utilize the sugar trehalose supplied by *Symbiodinium* at the developmental stage of the onset of symbiosis. In addition, this ability to metabolize trehalose persists into the adult stage and is present in at least three species of symbiotic cnidarians.

Importantly, trehalose has the characteristics of a reliable signal in this system in the sense that it is costly to the sender and transmits accurate information about its metabolic state. To generate the fixed carbon compound trehalose for use as a chemical cue, the symbiont’s photosynthetic ability must be intact and producing energy above its own metabolic needs. Given that the provision of fixed carbon from the symbiont to the cnidarian host is essential for maintenance of this symbiosis, this signal may allow the cnidarian to identify healthy symbionts. Amongst fixed carbon compounds, trehalose may be an ideal chemo-attractant because it is a non-reducing disaccharide containing a very stable α,α-1,1-glucoside bond between two glucose molecules, producing one of the most thermodynamically and kinetically stable non-reducing sugars [45].

### Chemo-attraction on the Reef

Understanding more about trehalose as a chemical cue on the reef is critical to understanding its biological relevance. In the laboratory, *F. scutaria* larvae demonstrated a clear preference for trehalose in the Y-maze assay. Moreover, the trehalose gradient stimulated the same changes in body morphology as when *F. scutaria* were taking up *Artemia* (brine shrimp) homogenate and *Symbiodinium* as described by Schwarz et al. [11]. While these Y-maze experiments do not mimic field conditions, as these types of perceptual experiments are difficult to conduct in complex environments, they indicate that coral larvae can specifically perceive trehalose and begin feeding, which is an essential step in the uptake of *Symbiodinium*.

There is some literature stating that larvae cannot use chemical cues for chemo-attraction over large distances because they cannot swim for long distances in a directional manner due to water flow in their environment, reviewed in [46]. However, recent studies disagree with these arguments. For example, Koehl and Hadfield have performed and reviewed a number of studies examining flow under field and laboratory conditions, and examined larval movement under various conditions and concluded that small larvae swim slowly but can alter their location in ambient water flow [46]. In addition, plannulae from three different *Acropora* species have recently been found to swim towards a preferred water source in a two-channel Atema plume [47] at a flow rate of 4.2 mm/s [26]. The overall flow rate in Kaneohe Bay is 10–20 cm/s [48] about 20 times the rate in the plume. However, the flow within the reef structure where the *F. scutaria* live would be much lower and conceivably within the flow rate measured in the Atema plume, suggesting that the larvae swimming toward a source would be well within the physical parameters of the system.

Other factors that support the hypothesis that *F. scutaria* can swim in a directional manner to follow chemical cues are related to the physiology of their eggs and the fine scale hydrodynamics of the reefs that *F. scutaria* inhabit in Kaneohe Bay. While the dispersal of *F. scutaria* larvae has not been described and the motility of adults prevents using their location as a proxy for larval settlement [49], it is clear that the adults live and breed in small holes and indentations on the reef, which provide a calm microenvironment, especially during reproduction in the summer months. *F. scutaria* eggs are negatively buoyant, which causes the developing embryos and resulting larvae to sink to the bottom in these calm microenvironments. Therefore, the larvae may not experience typical turbulent planktonic conditions. Additionally, water velocities within the reef have been shown to be relatively low with regions of both upward and downward flow, depending on the shape of the surface [50]. At sites other than Kaneohe Bay, *F. scutaria* has similarly been reported to be found in crevices on rubble substrata [49,51,52], which likely also give rise to calm microenvironments for the larvae formed after spawning. Taken together, these data suggest that there may be very low velocities in the immediate area of the *F. scutaria* larvae and the larvae may be able to detect cues in their environment and affect their position accordingly.

What trehalose concentration might be in the reef sediment and would it be high enough for the larvae to perceive? Free-living *Symbiodinium* reside only on reefs [55], [56], and, within the reef habitat, they are most concentrated within reef sediments (1000 to 4000 cells/ml) with lower densities (up to 80 cells/ml) in the water column [56]. Previous work has shown that larvae from the coral *Acropora monticulosa* acquire *Symbiodinium* earlier and at higher densities when they are incubated with reef sediment from the oxic layer compared to water from above the reef [53]. From our work on the concentration of trehalose released from *Symbiodinium* (e.g., 10^7^ cells/ml in 6 h) into seawater, we estimated that each cell released 7 × 10^−14^ moles of trehalose over that time. If we use the highest level of *Symbiodinium*/ml in the sediment, these numbers would produce 2.8 × 10^−7^ M trehalose in 1 ml of sediment over a 6 h period, assuming no loss by diffusion or bulk transport. This concentration is well within the range of sensitivities of many marine organisms, such as lobsters that have 10^−8^ M sensitivities [54]. However, there are several questions related to the robustness of this estimate. Diffusion is required to make the gradient and will lower the trehalose concentration in the sediment, but the rate of trehalose diffusion under these conditions is not known and likely varies substantially in different regions of the reef due to water flow and composition of sediment. In contrast, it is possible that this may be an underestimate of trehalose levels. It is not clear how deeply into the sediment Littman et al. sampled [56]. Takabayashi et al. [55] suggest that the oxic depth of the sediment is actually quite shallow (1–5mm), and given Littman et al.’s sampling methods, it is possible that the density of *Symbiodinium* in the oxic layer, where enough sunlight is available for photosynthesis, is actually much higher than reported. Furthermore, increases in local concentration of *Symbiodinium* from clumpy pellets released by sexually mature *Fungia* may play an important role in boosting levels of symbionts. Finally, it is not yet known when the trehalose might be perceived by the cnidarian larvae. Trehalose may act to orient the larvae towards the symbiont when they are in close proximity and then induce feeding behavior to facilitate uptake. Alternatively, it may be that trehalose plays a role in a more long-distance signaling cue of the type recently described [27], [28]. There may be only one role for trehalose, or the role may differ depending on the cnidarian species and the features of a particular reef.

Given the potentially small numbers of symbionts in the environment, the presence of sufficient adults containing healthy symbionts may be critical to seeding the reef during spawning to maintain and boost symbiont populations, thus allowing the symbionts and larvae to find one another as has been described in other systems [56,57]. Recent data demonstrates that some marine larvae demonstrate a preference towards water taken from live, healthy coral reefs [27], [28]. Taken together with our data, this suggests that there may be a positive feedback loop where healthy coral reefs recruit marine larvae which settle and sustain the reef. However, if the numbers of healthy animals on the reef decreases, there may be an insufficient signal for recruitment, leading to further degradation of the reef.

## Conclusions

We have found that in the symbiotic relationship between corals and *Symbiodinium*, fixed carbon in the form of trehalose produced by the symbiont plays a key role in how these partners find each other. This signal may yield information about the nutritional status and potential fitness of the signal generator and may serve the critical role of attracting the partners towards one another, where further information, in direct cell-cell interactions, can then be exchanged. Given that a likely benefit of horizontal infection is the identification of symbionts best suited to a particular environment, the trehalose cue, reflecting photosynthetic capability, may be particularly important to a potential host.

Symbiosis between coral and intracellular dinoflagellates is critical for coral viability and reef ecosystem productivity. Most larval coral take up symbionts from their environment; but how these symbiotic partners signal and sense one another has been unknown. To help solve this mystery, we demonstrated in *F. scutaria* coral and their *Symbiodinium*: 1) a release of the disaccharide trehalose into the environment by the symbionts, 2) a coral larval chemo-attraction and feeding response in the presence of trehalose, 3) the presence of the enzyme, trehalase, in coral tissue, but not in the symbionts, and 4) adult release of live *Symbiodinium*, thus seeding the reef. To our knowledge, this is the first report of a specific chemical cue attracting the motile coral larvae to the symbiont.

## Supporting Information

S1 Fig Chemo-orienting Behavior: *F. scutaria* larvae responding to FSW in both arms of the Y-maze. Larvae were added to the base of the Y-maze, 0.5 μl of FSW was added to at the tops of the arms, and then the larvae were allowed to swim around for 3 min. This video shows the last 10 sec of the run, demonstrating a lack of chemo-orienting behavior in either arm.
(MOV)

S2 Fig Chemo-orienting Behavior: *F. scutaria* larvae responding to 0.1 M trehalose in the upper arm of the Y-maze. Larvae were added to the base of the Y-maze, 0.5 μl of FSW was added to the top of the lower arm and 0.5 μl of 0.1 M trehalose was added to the top of the upper arm, and then the larvae were allowed to swim around for 3 min. This video shows the last 10 sec of the run, demonstrating clear chemo-orienting behavior of the larvae in the upper arm in response to added trehalose.
(MOV)

S3 Fig Identification of potential trehalase-encoding genes in a coral: the scleractinian coral *Acropora digitifera* contains a gene predicted to encode trehalase. *D. melanogaster* trehalase enzyme (AAM68193.1) and *A. digitifera* (aug_v2a.15372) aligned using ClustalW. Black boxes, identical residues; gray boxes, similar residues. The *D. melanogaster* gene is known to encode an enzyme with trehalase activity. Regions with identity to the Prosite trehalase PDOC00717 signature 1 (PGGRFxExYxWDxY)and signature 2 (QWDxPx{GAV}W{PAS}P) are denoted by asterisks above the sequence.
(TIFF)

S4 Fig Identification of potential trehalase-encoding gene in the transcriptomes of *F. scutaria*. BLAST search of the transcriptome of *F. scutaria* (http://people.oregonstate.edu/~meyere/data.html) identified a likely trehalase. This 243-nucleotide sequence had an open reading frame throughout its entire length (81 amino acids). The *F. scutaria* predicted protein was 79% identical to the *A. digitifera* protein. The homology extended through the entire length of the *F. scutaria* open reading frame but did not reach the regions with the signature motifs for the trehalase enzyme. The numbers above the sequence correspond to the amino acid position in the *A. digitifera* sequence. Black boxes, identical residues; gray boxes, similar residues.
(TIF)
