# Supplementary figures and images for: Trehalose Is a Chemical Attractant in the Establishment of Coral Symbiosis
Source: PLoS One. 2015 Jan 28;10(1):e0117087. doi: 10.1371/journal.pone.0117087 (PMC4309597; doi:10.1371/journal.pone.0117087)

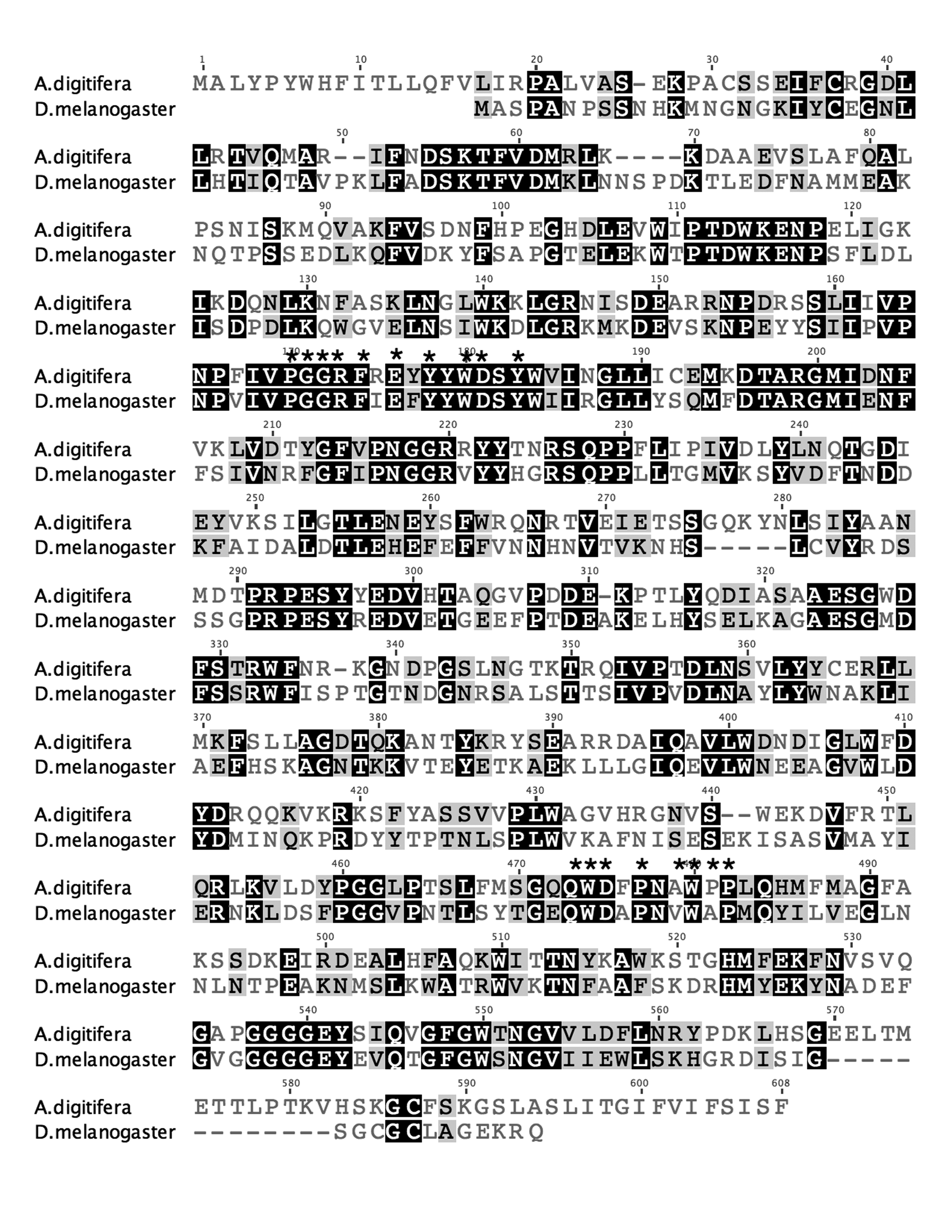

Supplement: S3 Fig — D. melanogaster trehalase enzyme (AAM68193.1) and A. digitifera (aug_v2a.15372) aligned using ClustalW. Black boxes, identical residues; gray boxes, similar residues. The D. melanogaster gene is known to encode an enzyme with trehalase activity. Regions with identity to the Prosite trehalase PDOC00717 signature 1 (PGGRFxExYxWDxY)and signature 2 (QWDxPx{GAV}W{PAS}P) are denoted by asterisks above the sequence. (TIFF) [file pone.0117087.s003.tiff]

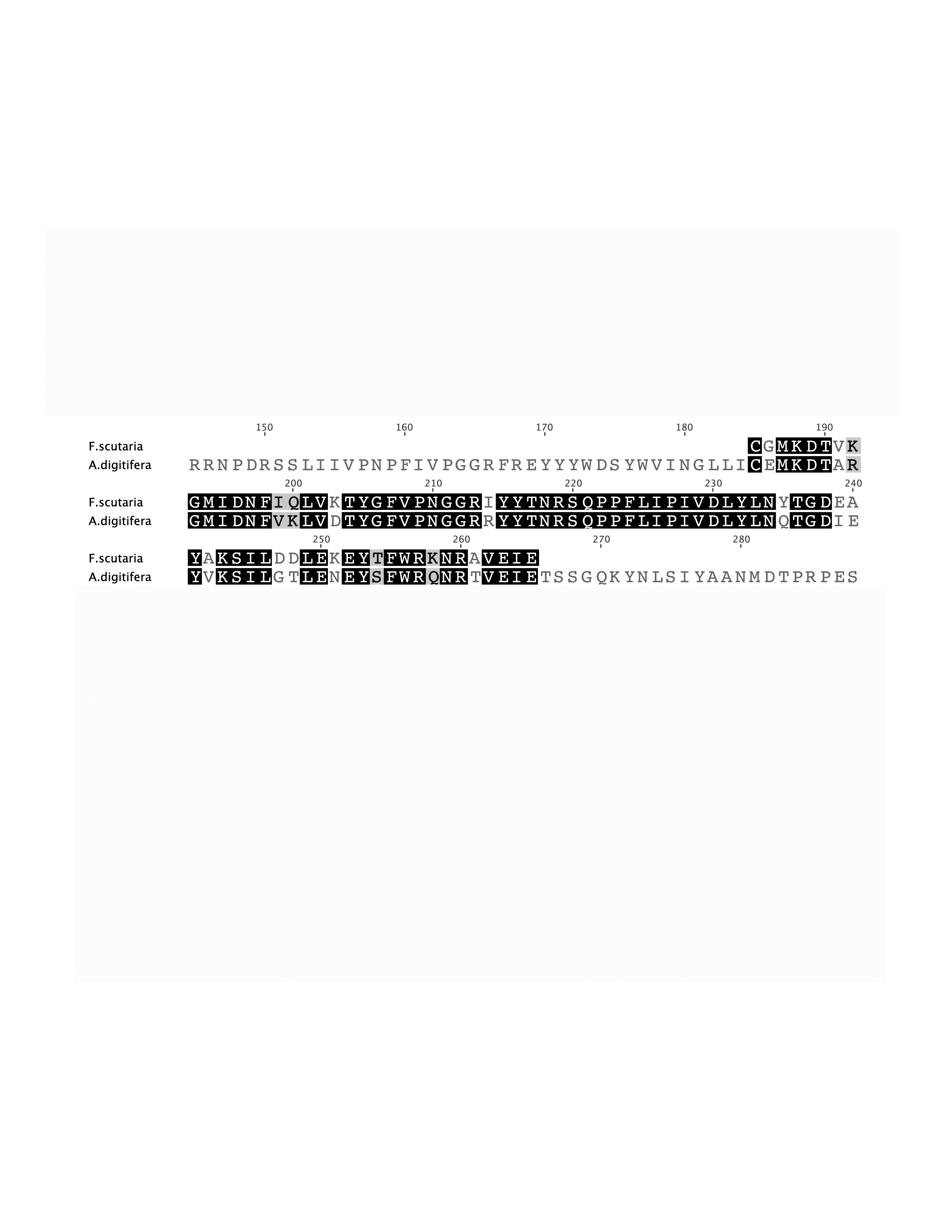

Supplement: S4 Fig — BLAST search of the transcriptome of F. scutaria (http://people.oregonstate.edu/~meyere/data.html) identified a likely trehalase. This 243-nucleotide sequence had an open reading frame throughout its entire length (81 amino acids). The F. scutaria predicted protein was 79% identical to the A. digitifera protein. The homology extended through the entire length of the F. scutaria open reading frame but did not reach the regions with the signature motifs for the trehalase enzyme. The numbers above the sequence correspond to the amino acid position in the A. digitifera sequence. Black boxes, identical residues; gray boxes, similar residues. (TIF) [file pone.0117087.s004.tif]
